# Coronary Artery Vasospasm: Cellular and Molecular Insights

**DOI:** 10.3390/cells15131145

**Published:** 2026-06-24

**Authors:** Stefan Juricic, Milan Dobric, Sinisa Stojkovic, Milorad Tesic, Ivana Jovanovic, Marko Banovic, Ratko Lasica, Srdjan Aleksandric, Ana Perunicic, Jovana Klac, Dejan M. Lazovic, Filip Simeunovic, Sashko Nikolov, Olga Petrovic, Dejan Simeunovic

**Affiliations:** 1 Clinic of Cardiology, University Clinical Center of Serbia, 11000 Belgrade, Serbia; 2 Faculty of Medicine, University of Belgrade, 11000 Belgrade, Serbia; iatros007@gmail.com (M.D.);; 3 Dedinje Cardiovascular Institute, 11040 Belgrade, Serbia; 4 Department of Cardiology, Emergency Center, University Clinical Center of Serbia, 11000 Belgrade, Serbia; 5 Clinic for Cardiac Surgery, University Clinical Center of Serbia, 11000 Belgrade, Serbia; 6 Faculty of Medical Sciences, Goce Delcev University, 2000 Stip, North Macedonia

**Keywords:** coronary artery vasospasm, vascular smooth muscle cells, Rho-kinase, endothelial dysfunction, microvascular dysfunction, perivascular adipose tissue, chronic inflammation

## Abstract

Coronary artery vasospasm (CAV) is a transient, reversible constriction of the epicardial coronary arteries that reduces coronary blood flow and may cause myocardial ischemia. Despite its clinical significance, CAV remains underdiagnosed and can present as chest pain, acute coronary syndrome, malignant arrhythmias or sudden cardiac death. Vasospasm may occur in both angiographically normal coronary arteries and at sites of pre-existing atherosclerotic stenosis. The pathophysiology of CAV is multifactorial and involves vascular smooth muscle cells (VSMCs) hyperreactivity, endothelial dysfunction, chronic inflammation and autonomic dysregulation. VSMCs contraction is mediated by phosphorylation of the myosin light chain (MLC) through calcium (Ca^2+^)/calmodulin-dependent myosin light chain kinase (MLCK), while relaxation is regulated by myosin light chain phosphatase (MLCP). Increased intracellular Ca^2+^ levels and enhanced Ca^2+^ sensitivity contribute to excessive vasoconstriction. Rho-kinase (ROCK) plays a pivotal role in sustained vasospasm by inhibiting MLCP, thereby promoting prolonged smooth muscle contraction. Endothelial dysfunction contributes to CAV by disrupting normal vascular tone regulation, largely as a result of decreased nitric oxide (NO) mediated vasodilation. Chronic low-grade inflammation and oxidative stress exacerbate both endothelial dysfunction and VSMCs contraction. Understanding these molecular mechanisms is essential for identifying novel therapeutic targets. Emerging treatment strategies, including ROCK inhibitors, endothelin receptor antagonists and anti-inflammatory agents, may improve outcomes in patients with refractory CAV.

## 1. Introduction

Cardiovascular diseases, particularly ischemic heart disease, remain the leading cause of mortality worldwide and represent one of the major contributors to global disability [1]. Coronary artery vasospasm (CAV) is an important cause of myocardial ischemia characterized by a transient, reversible constriction of the epicardial coronary arteries [2]. As a leading cause of coronary vasomotor dysfunction, CAV constitutes a principal endotype of ischemia with non-obstructive coronary arteries (INOCA) [3]. This condition carries immense clinical and socioeconomic significance, given that a substantial proportion of patients with suspected coronary artery disease (CAD) undergo invasive coronary angiography that reveals normal or non-obstructive coronary arteries [4]. Consequently, managing these patients frequently necessitates advanced diagnostic strategies, which are often unavailable in lower-volume centers, thereby placing a considerable burden on healthcare systems.

Clinically, CAV presents with a broad spectrum of manifestations, ranging from chronic stable angina and acute coronary syndromes to malignant ventricular arrhythmias and sudden cardiac death. Thus, it encompasses the entire clinical continuum from angina with non-obstructive coronary arteries (ANOCA) to myocardial infarction with non-obstructive coronary arteries (MINOCA).

Insufficient awareness and education regarding CAV, together with the limited use of diagnostic testing in routine clinical practice, are among the main reasons why the true prevalence of this condition remains unknown [5]. Furthermore, significant heterogeneity in institutional evaluation protocols and varying selection criteria for patients referred for invasive provocative testing significantly contribute to inconsistent epidemiological data across historical cohorts.

It has been clearly demonstrated that CAV is more common among Asian patients than among Caucasian patients [6,7]. Furthermore, epicardial spasm occurs more frequently in men, in whom smoking is a more prevalent risk factor, whereas women appear to be more prone to developing microvascular dysfunction [8,9].

Nevertheless, recent systematic reviews and meta-analyses have provided robust insights into the prevalence and demographics of this population. Pooled data from large-scale analyses indicate a remarkably consistent prevalence of approximately 40–43% for epicardial spasm and 24–25% for microvascular spasm among ANOCA cohorts, with an average patient age of 58.2 years and women accounting for nearly half (44%) of the affected population [10,11].

**Figure 1:**
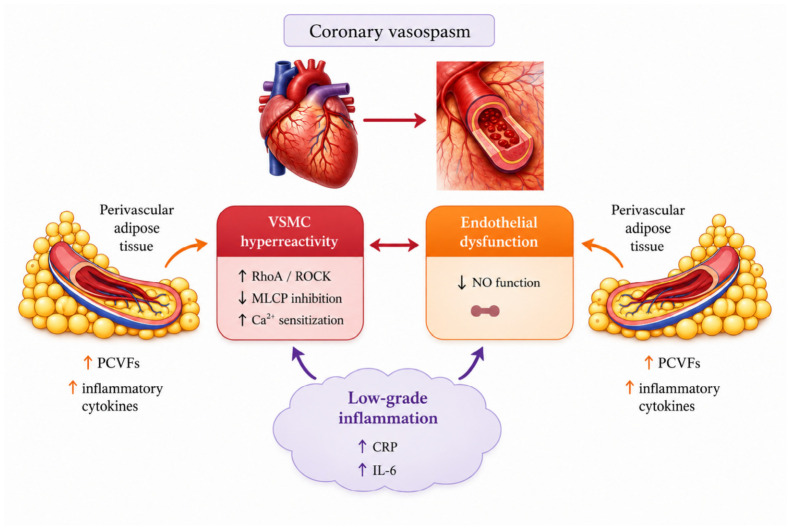
Mechanisms underlying coronary vasospasm. Coronary vasospasm arises from the interaction between endothelial dysfunction and VSMCs contraction, driven by reduced NO bioavailability and enhanced RhoA/ROCK signaling with increased Ca^2+^ sensitivity. Perivascular adipose tissue (PVAT)-derived inflammatory cytokines and perivascular contractile factors (PCVFs), together with low-grade inflammation (elevated C-reactive protein (CRP) and Interleukin-6 (IL-6), further amplify vasoconstriction. VSMCs—vascular smooth muscle cells, RhoA—Ras homolog family member A, ROCK—Rho-associated protein kinase, MLCP—myosin light chain phosphatase, Ca^2+^—calcium, NO—nitric oxide, PVAT—perivascular adipose tissue, PCVFs—perivascular contractile factors, CRP—C-reactive protein, IL-6—interleukin-6.

Several mechanisms have been implicated in the pathogenesis of CAV, including VSMC contraction, endothelial dysfunction, oxidative stress and inflammation, often precipitated by established cardiovascular risk factors such as hypertension, hypercholesterolemia, diabetes mellitus, obesity and smoking [12]. Autonomic dysregulation and genetic predisposition have also been shown to contribute to the development of CAV [13]. However, it remains difficult to determine whether any single mechanism plays a dominant causal role [14] [Figure 1].

The primary aim of this narrative review was to describe the pathophysiological mechanisms underlying both epicardial and microvascular coronary vasospasm. A literature search was conducted using the PubMed and Google Scholar databases. The search strategy included the following keywords and their combinations: coronary artery vasospasm; vasospastic angina; microvascular spasm; coronary microvascular dysfunction; endothelial dysfunction; nitric oxide; NO dysfunction; vascular smooth muscle cells; Rho-kinase; RhoA; myosin light chain; calcium sensitization; perivascular adipose tissue; chronic inflammation; and oxidative stress. Boolean operators (AND or OR) were used to combine search terms and to improve search strategy.

The search encompassed studies published between 1985 and 2026. Earlier studies were included to capture the foundational research that established the basis and key concepts that to this day remain central to the current understanding of the pathophysiological mechanisms of CAV, including endothelial dysfunction, nitric oxide signaling and vascular smooth muscle hyperreactivity. The most recent publications were incorporated to reflect emerging evidence and advances in the field.

**Figure 2:**
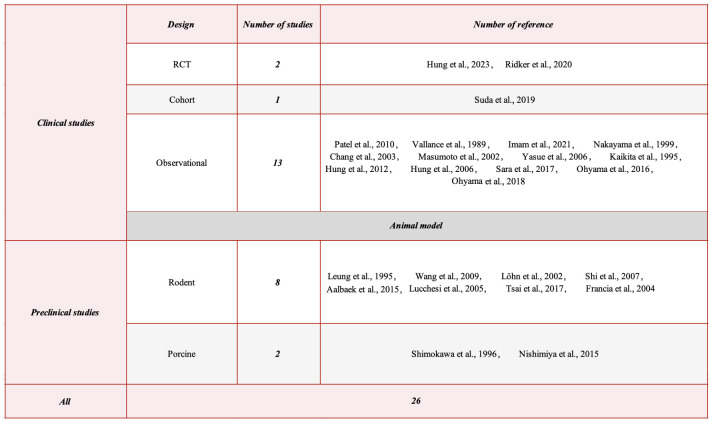
Classification of Studies According to Study Design and Experimental Model. Summation of the included studies, categorized into clinical and preclinical investigations according to study design, experimental model, number of studies and corresponding reference numbers. RCTs: [15,16]; cohort studies: [17]; observational studies: [4,18,19,20,21,22,23,24,25,26,27,28,29]; rodent studies: [30,31,32,33,34,35,36,37]; porcine studies: [38,39].

Only studies published in English were considered. Original research articles and review articles constituted the majority of included references. The review included both clinical and preclinical studies, including randomized controlled studies and cohort studies. Animal studies involved rodent and porcine models and clinical studies included relevant human populations [Figure 2].

## 2. Endothelial Dysfunction

The vascular endothelium plays a central role in regulating vascular tone and smooth muscle proliferation through the balanced release of vasodilators and vasoconstrictors. It also modulates hemostasis, inflammatory responses and leukocyte adhesion and infiltration.

NO is considered the most important mediator in the development of CAV in the context of endothelial dysfunction. Coronary vasospasm can be provoked by agents such as acetylcholine, serotonin, ergonovine, or histamine. When the endothelium is intact, acetylcholine induces vasodilation through NO release. However, when the endothelium is damaged, acetylcholine directly stimulates VSMCs, resulting in vasoconstriction.

Endothelial nitric oxide synthase (eNOS) catalyzes the production of NO from L-arginine [40]. NO maintains basal vascular tone in both animals and humans [41,42] Impaired NO bioavailability has been strongly implicated in the pathogenesis of coronary vasospasm.

Patients with CAV have been shown to exhibit NO resistance at the level of platelet aggregation, reflecting impaired NO signaling [19].

NO dysfunction may occur at multiple levels, including reduced synthesis and NO resistance due to impaired function of its receptor, the heme-containing enzyme soluble guanylate cyclase (sGC).

An increase in intracellular Ca^2+^ concentration within endothelial cells leads to Ca^2+^ binding to calmodulin, which activates eNOS and stimulates NO production from L-arginine. sGC serves as the intracellular receptor for NO and catalyzes the conversion of guanosine 5′-triphosphate (GTP) into cyclic guanosine 3′,5′-monophosphate (cGMP). Binding of NO to sGC activates the enzyme, increasing intracellular cGMP levels. This triggers downstream signaling through cGMP-dependent protein kinase (PKG), cGMP-gated cation channels and cGMP-regulated phosphodiesterases. PKG phosphorylates the inositol 1,4,5-triphosphate receptor, leading to reduced intracellular Ca^2+^ concentration and smooth muscle relaxation [43].

Although NO has been reported to directly activate Ca^2+^-dependent potassium channels in VSMCs, this effect appears to require supraphysiological NO concentrations not typically observed in vivo [44].

Compelling evidence links CAV to genetic variations in the NOS3 gene, including the T-786-C mutation and polymorphisms such as NOS4a and Glu298Asp [20,21].

Multiple enzymatic systems within endothelial cells generate superoxide anions, including xanthine oxidase, cyclooxygenase, NADPH oxidase (NOX) and eNOS itself when uncoupled due to tetrahydrobiopterin deficiency or substrate depletion. Superoxide anions degrade NO into peroxynitrite, thereby reducing NO bioavailability [45].

Increased arginase activity, which shares L-arginine as a substrate with eNOS, has been shown to rise with aging, leading to reduced NO production and endothelial dysfunction. Aging is also associated with increased oxidative stress and reduced eNOS activity, further diminishing NO bioavailability [46].

Hypercholesterolemia exerts similar effects by enhancing superoxide production and decreasing NO bioavailability. In addition, elevated circulating levels of asymmetric dimethylarginine, an endogenous eNOS inhibitor, further impair NO synthesis [46].

## 3. Vascular Smooth Muscle Cells Hyperreactivity

**Figure 3:**
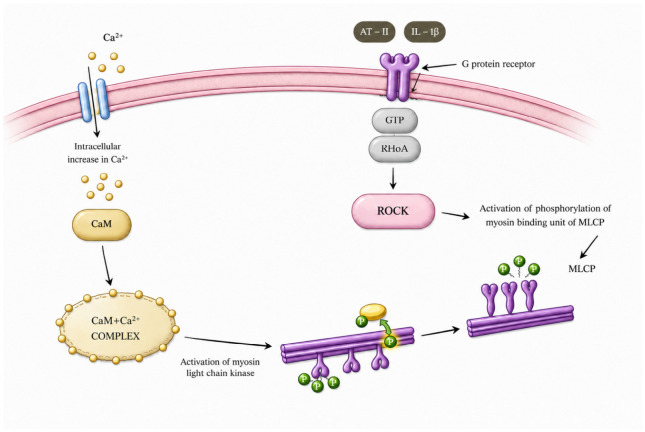
Schematic representation of VSMCs contraction regulated by Ca^2+^-dependent and RhoA/ROCK-mediated pathways. Intracellular Ca^2+^ influx promotes calmodulin activation and formation of the Ca^2+^–calmodulin complex, which activates MLCK, leading to phosphorylation of MLC and contraction. In parallel, G protein–coupled receptor stimulation by vasoactive and inflammatory mediators (e.g., Ang II, IL-1β) activates RhoA/ROCK signaling, which inhibits MLCP via phosphorylation of its myosin-binding subunit, thereby enhancing Ca^2+^ sensitization and sustaining contraction. Ca^2+^—Calcium, CaM—Calmodulin, MLC—Myosin light chain, MLCK—Myosin light chain kinase, MLCP—Myosin light chain phosphatase, ROCK—Rho-associated coiled-coil containing protein kinase, RhoA—Ras homolog family member A, GTP—Guanosine triphosphate, Ang II—Angiotensin II, IL-1β—Interleukin-1 beta.

An increase in intracellular Ca^2+^ concentration in VSMCs leads to Ca^2+^ binding to calmodulin, forming the Ca^2+^-calmodulin complex. This complex activates MLCK, which phosphorylates the MLC. The degree of MLC phosphorylation is a critical determinant of smooth muscle contraction [47] [Figure 3].

VSMCs contraction is regulated by the activation of actin–myosin cross-bridges through phosphorylation of MLC. MLC is phosphorylated by Ca^2+^/calmodulin-dependent MLCK and dephosphorylated by MLCP. The dynamic balance between MLCK and MLCP activity determines the contractile state of the VSMCs [47].

Both increased Ca^2+^ influx and enhanced Ca^2+^ sensitivity of contractile proteins contribute to VSMCs hypercontraction and are considered key mechanisms underlying CAV [47,48].

A central mediator of sustained CAV is ROCK, which enhances VSMCs contraction by inhibiting MLCP. ROCK phosphorylates the myosin-binding subunit of MLCP, thereby reducing phosphatase activity and promoting sustained MLC phosphorylation [22].

ROCK is a serine/threonine kinase with two isoforms encoded by distinct genes: ROCK1 (ROCKβ), located on chromosome 18 and encoding a 1345-amino acid protein [49], and ROCK2 (ROCKα), located on chromosome 12 and encoding a 1388-amino acid protein [30,50]. Both isoforms are expressed in VSMCs [51].

Although ROCK1 and ROCK2 share structural similarities, they regulate MLCP and vascular contractility through distinct mechanisms. ROCK2 appears to play a predominant role in modulating contractility in VSMCs [31].

The expression and activity of ROCK isoforms are modulated by inflammatory mediators such as angiotensin II (Ang II) and interleukin-1β (IL-1β) [52]. In vitro studies have demonstrated that nicotine potentiates the stimulatory effect of Ang II on ROCK expression, whereas estrogen exerts an inhibitory effect [53]. These findings may partially explain the higher incidence of vasospastic angina in postmenopausal women and smokers [53].

ROCK regulates MLC phosphorylation through two principal mechanisms: inactivation of MLCP via phosphorylation of its myosin-binding subunit, leading to Ca^2+^ sensitization and direct phosphorylation of MLC. Experimental studies indicate that inhibition of MLCP is the dominant mechanism responsible for Ca^2+^ sensitization in smooth muscle [54,55].

Shimokawa and colleagues developed porcine models of coronary vasospasm and demonstrated enhanced ROCK activity in coronary smooth muscle at spastic sites, further supporting its central role in the pathogenesis of CAV [14].

## 4. RhoA/ROCK Pathway in Endothelial Cells

The RhoA/ROCK pathway is not restricted to VMSC, but is also active in endothelial cells. Increased ROCK activity has been shown to reduce NO production and impair endothelial function [56,57].

**Figure 4:**
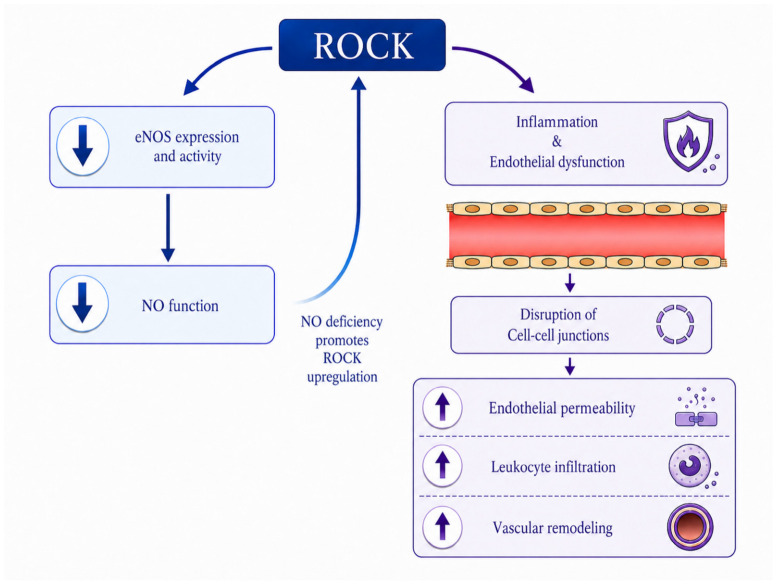
ROCK-mediated endothelial dysfunction. Increased ROCK activity reduces endothelial nitric oxide synthase (eNOS) expression and activity, leading to impaired nitric oxide (NO) bioavailability and function. In turn, NO deficiency further promotes ROCK upregulation, creating a vicious cycle. Simultaneously, ROCK contributes to inflammation and endothelial dysfunction, disrupts endothelial cell-to-cell junctions, increases endothelial permeability, promotes leukocyte infiltration and stimulates vascular remodeling. ROCK—Rho-kinase, eNOS—endothelial nitric oxide synthase, NO—nitric oxide.

Mechanistically, ROCK decreases endothelial NO bioavailability by reducing eNOS expression and activity, while simultaneously increasing endothelial contractility through actomyosin tension. This exact mechanism of action is disruption of cell-to-cell junctions which increases endothelial permeability, facilitates leukocyte infiltration and triggers pro-inflammatory signaling, thereby driving adverse vascular remodeling [58] [Figure 4].

In human endothelial cells in vitro, activation of Rho-kinase has been shown to reduce eNOS expression and activity, an effect that was reversed by the Rho-kinase inhibitor hydroxyfasudil. This finding supports the concept that ROCK activation directly contributes to endothelial dysfunction by suppressing the endothelial NO pathway [59].

Importantly, decreased endothelial NO bioavailability may further increase RhoA/ROCK activity in the coronary arteries, suggesting a reciprocal regulatory relationship between NO signaling and ROCK activation [15,60,61,62,63]. Under physiological conditions, NO suppresses RhoA expression through the cGMP-dependent protein kinase G pathway. Conversely, NO deficiency promotes ROCK upregulation, thereby enhancing vasoconstriction and vascular hyperreactivity [61].

Clinical studies have also demonstrated that ROCK inhibition improves endothelial function in patients with coronary artery disease, further supporting the interaction between impaired NO signaling and increased ROCK activity [15].

Angiotensin II activates the ROCK2 isoform in endothelial cells, leading to impaired endothelium-dependent relaxation. In experimental mouse models, inhibition of this isoform has been shown to restore endothelial function, further supporting the role of endothelial ROCK2 activation in angiotensin II-induced endothelial dysfunction [64].

Oxidized LDL has also been shown to activate the RhoA/ROCK pathway and to potentiate angiotensin II-induced vasoconstriction [65]. ROCK is also an important downstream target of 3-hydroxy-3-methylglutaryl coenzyme A reductase inhibitors, commonly known as statins. Inhibition of the Rho pathway by statins improves eNOS expression by stabilizing eNOS mRNA and stimulates eNOS activity through activation of the phosphatidylinositol 3-kinase/Akt pathway. These pleiotropic effects may contribute to improved endothelial function and cardiovascular protection [66,67,68].

Inflammatory mechanisms may additionally contribute to ROCK upregulation. In a porcine model, chronic focal application of interleukin-1β induced intimal lesions at the injection site. Subsequent intracoronary administration of serotonin or histamine provoked focal coronary vasospasm, emphasizing the role of inflammation in promoting vasospastic responses [38].

## 5. Inflammation

Chronic low-grade inflammation is strongly associated with CAV. Increasing evidence suggests that inflammatory processes play an important role in the pathogenesis of coronary vasomotor abnormalities by affecting both endothelial function and VSMC reactivity. Several experimental and clinical studies have demonstrated a link between vascular inflammation and enhanced coronary vasoconstriction. Smoking, which represents one of the major risk factors for CAV, is also associated with persistent low-grade inflammation and a prothrombotic state [23,39,69].

Numerous inflammatory biomarkers have been reported to be elevated in patients with CAV, including high-sensitivity C-reactive protein (CRP), interleukin-6 (IL-6), soluble CD40 ligand, P-selectin, monocyte chemoattractant protein-1 (MCP-1), soluble intercellular adhesion molecule-1 (sICAM-1) and soluble vascular cell adhesion molecule-1 (sVCAM-1) [24,70,71,72]. These biomarkers reflect endothelial activation, platelet activation, and leukocyte recruitment, all of which contribute to vascular dysfunction.

At the cellular level, elevated MCP-1 may promote monocyte recruitment and activation within the vascular wall, while increased sICAM-1 and sVCAM-1 levels reflect endothelial activation and enhanced leukocyte-endothelial adhesion. This consequently leads to reduced NO bioavailability, increased oxidative stress and enhanced VSMC contraction [25,26].

**Figure 5:**
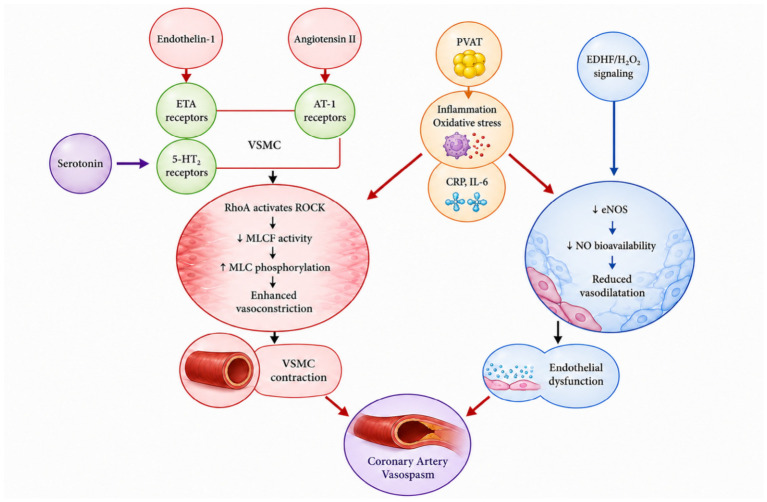
Interplay Between VSMC Contraction, Endothelial Dysfunction and Inflammation in Coronary Vasospasm. Vasoconstrictor stimuli, including endothelin-1, angiotensin II and serotonin, activate ETA, AT1 and 5-HT_2_ receptors on vascular smooth muscle cell (VSMC), leading to RhoA/ROCK pathway activation, inhibition of myosin light chain phosphatase (MLCP) and myosin light chain (MLC) phosphorylation, resulting in enhanced VSMC contraction. Inflammation, oxidative stress, elevated CRP and IL-6 levels, perivascular adipose tissue (PVAT) dysfunction and EDHF/H_2_O_2_ signaling contribute to endothelial dysfunction by reducing eNOS activity and nitric oxide (NO) bioavailability, as well as in VMSC contraction. The combined effects of enhanced VSMC contraction and endothelial dysfunction promote coronary artery vasospasm. VSMC- vascular smooth muscle cell; ROCK- Rho kinase, MLCP- myosin light chain phosphatase; MLC- myosin light chain; PVAT- perivascular adipose tissue; CRP- C-reactive protein; IL-6- interleukin-6; eNOS- endothelial nitric oxide synthase; NO- nitric oxide; EDHF- endothelium-derived hyperpolarizing factor, H_2_O_2_- hydrogen peroxide.

Rho-kinase activity is increased by inflammatory stimuli, such as angiotensin II and IL-1β, in VSMCs. Rho-kinase expression, both at the mRNA and protein levels, as well as its activity, is upregulated by the above-mentioned inflammatory stimuli through mechanisms mediated by protein kinase C (PKC) and nuclear factor-κB (NF-κB) [Figure 5].

**Figure 6:**
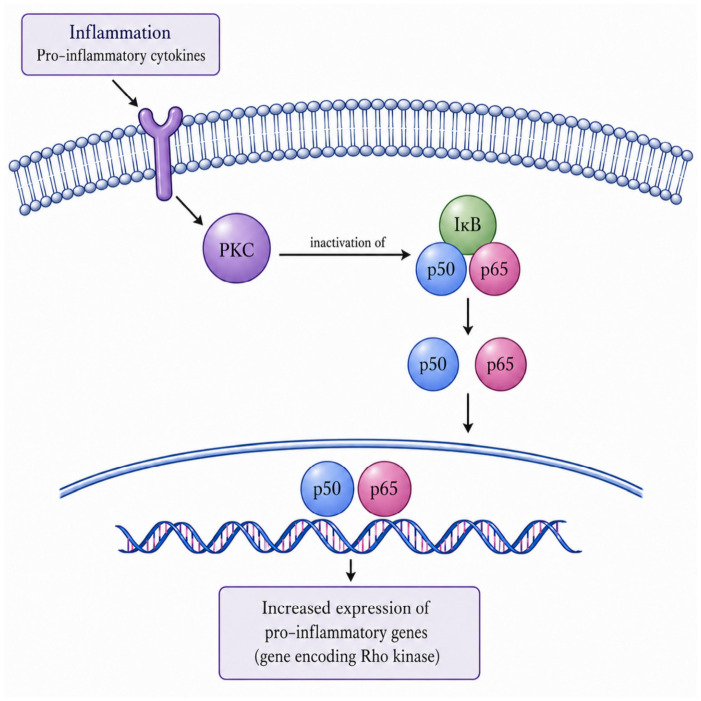
Inflammation-mediated mechanisms leading to endothelial dysfunction. In inflammation, pro-inflammatory cytokines trigger pro-inflammatory gene expression, including genes encoding Rho-kinase. Pro-inflammatory cytokines bind to membrane receptors and activate intracellular signaling pathways involving protein kinase C (PKC). PKC-mediated signaling leads to the inactivation and degradation of IκB, which normally retains the NF-κB p50/p65 complex in an inactive state. Following IκB inactivation, the p50/p65 NF-κB heterodimer is released and translocates into the nucleus, where it promotes transcription of pro-inflammatory genes. NF-κB—nuclear factor κB, IκB—inhibitor of nuclear factor κ B, PKC—protein kinase C, p50—NF-κB p50 subunit, p65—NF-κB p65 subunit, DNA—deoxyribonucleic acid, ROCK—Rho-kinase.

Through PKC-mediated degradation and inactivation of inhibitor κB (IκB), NF-κB is activated. This activation involves the p50 and p65 subunits, which represent the most common NF-κB heterodimers. Following activation, NF-κB translocates from the cytoplasm to the nucleus, primarily through the p65-containing heterodimer, where it binds to target DNA sequences. This results in increased expression of various pro-inflammatory genes, including the gene encoding Rho-kinase. Since increased Rho-kinase expression further enhances the sensitivity of signaling pathways activated by inflammatory agonists, this creates a vicious cycle in which inflammation and Rho-kinase activation mutually reinforce each other [73] [Figure 6].

Elevated levels of CRP, a systemic inflammatory mediator, are associated with endothelial dysfunction. CRP decreases eNOS and prostacyclin synthase expression, which consequently reduces the vasodilatory capacity of the endothelium [74,75]. This impairment of endothelium-dependent vasodilation creates a pro-constrictive vascular environment.

Furthermore, CRP has been identified as a marker of exaggerated coronary vasoreactivity in patients with non-obstructive CAD [27]. Higher CRP levels have also been correlated with increased mortality in patients with coronary vasospasm [16]. Taken together, these findings highlight the important role of inflammatory pathways in the development and progression of coronary vasospasm.

## 6. Perivascular Adipose Tissue

A specialized cellular population surrounding blood vessels is known as PVAT. PVAT is phenotypically distinct from other adipose tissue depots and produces numerous vasoactive factors that exert paracrine effects on vascular function [76]. Under physiological conditions, PVAT contributes to vascular homeostasis by releasing factors that regulate vascular tone and vascular remodeling.

However, in the presence of cardiovascular risk factors such as obesity or diabetes mellitus, PVAT dysfunction develops due to changes in adipose tissue volume and composition, which subsequently alters the expression of vasoactive mediators [12]. These alterations promote a shift toward a pro-inflammatory and vasoconstrictive vascular environment.

Using coronary CT angiography, Ohyama et al. demonstrated that patients with vasospastic angina have a significantly greater volume of PVAT surrounding the spastic segments of coronary arteries [28]. This finding suggests that PVAT may play an active role in the pathogenesis of coronary vasospasm.

Vasoactive factors released by PVAT play an important role in the regulation of vascular tone, arterial growth and vascular remodeling [77]. These mediators are generally classified into perivascular relaxing factors and PCVFs.

Among the perivascular relaxing factors, the first to be shown to be released from PVAT and transported to the vascular wall to exert a paracrine vasodilatory effect was the adipocyte-derived relaxing factor [32]. The most well-known adipokines with vasodilatory properties include adiponectin and leptin, although several others such as omentin, visfatin, apelin and irisin have also been identified, while their vascular effects remain less clearly defined [78].

Some of the most recognized adipocyte-derived relaxing factors include reactive oxygen species (ROS) such as hydrogen peroxide and hydrogen sulfide, which are capable of modulating vascular tone [33].

The most accurate method for detecting increased inflammation within PVAT is 18F-fluorodeoxyglucose positron emission tomography/computed tomography (18F-FDG PET/CT), as this imaging modality reflects increased glucose metabolic activity associated with inflammatory processes. Studies have shown that coronary perivascular FDG uptake is significantly increased in patients with coronary vasospasm [29].

The same study also demonstrated increased ROCK activity in circulating leukocytes in patients with increased PVAT volume and coronary vasospasm, suggesting a potential mechanistic link between PVAT inflammation and enhanced VSMCs contractility.

Inflammatory cytokines secreted from PVAT also play an important role in the development of coronary vasospasm. Experimental studies have demonstrated that adventitial application of IL-1β in pigs with preserved endothelial function induces coronary vasospasm in vivo through increased ROCK activity and subsequent inhibition of MLCP. This mechanism leads to enhanced Ca^2+^ sensitivity of VSMCs and promotes vasoconstriction [38].

Early pathological observations also support the role of adventitial inflammation in coronary vasospasm. Autopsy findings in patients with coronary artery spasm demonstrated accumulation of mast cells within the adventitia of coronary arteries [79]. In the context of contemporary knowledge regarding cellular and molecular mechanisms of vascular inflammation, these findings suggest that inflamed PVAT may significantly contribute to the pathogenesis of CAV.

Under physiological conditions, PVAT exerts an anticontractile effect on the underlying vascular wall. Experimental studies have shown that PVAT decreases Ca^2+^ sensitivity of VSMCs, thereby reducing vasoconstriction in healthy rat coronary arteries [34]. However, when adventitial inflammation develops, signaling pathways within PVAT are altered. Increased secretion of inflammatory cytokines leads to activation of the ROCK pathway and suppression of MLCP activity, resulting in enhanced VSMCs contraction and coronary vasospasm [38].

This mechanism of vasospasm development is known as the “outside-in” signaling hypothesis, in which inflammatory and metabolic signals originating from the adventitia and PVAT propagate inward toward the medial layer of the vessel wall, ultimately leading to VSMC contraction and coronary artery spasm [38].

Therefore, PVAT represents an important interface linking metabolic disturbances, vascular inflammation and VSMC contraction in the pathogenesis of coronary vasospasm.

## 7. Autonomic Nervous System

Among other pathophysiological mechanisms, it has been confirmed that autonomic dysfunction can lead to the development of CAV. Paradoxically, CAV can be precipitated by both an acute increase in sympathetic tone and an elevation in parasympathetic activity [13].

### 7.1. Sympathetic Activity

Noradrenaline, the primary neurotransmitter of efferent sympathetic fibers, induces vasoconstriction by stimulating α- adrenergic receptors on vascular smooth muscle cells (VSMCs). Clinical observations show that CAV can be precipitated by exogenous catecholamines or physiological stressors like exercise, which increase sympathetic outflow [80,81].

Additionally, substances such as cocaine and amphetamines provoke direct VSMC contraction through α- adrenergic pathways and increase the baseline sensitivity of these cells to circulating catecholamines [82].

### 7.2. Vagal Activity

Acetylcholine (ACh) is the main neurotransmitter of the parasympathetic system [83].

It binds to two families of receptors: muscarinic (M) receptors, which belong to the G-protein family and nicotinic (n) receptors, which function as ligand-dependent ion channels. The muscarinic family consists of 5 subtypes, each characterized by a different function. M receptors are widely distributed throughout the conduction system, cardiomyocytes and coronary arteries [84,85].

The M2 subtype is located mainly in the sinoatrial and atrioventricular nodes, through which ACh regulates the resting heart rate and improves heart rate recovery after physical activity. Some studies have shown that ACh acts as a cardioprotector and antagonist to ischemia; specifically, M3 receptors interfere with the production of reactive oxygen species (ROS), preserve mitochondrial function and reduce the size of myocardial injury [84].

In addition, M3 receptors predominantly mediate the effect of ACh on the endothelium and VSMCs by being coupled with Gq proteins, inducing the activation of endothelial phosphoinositol-specific phospholipase C and the consequent formation of inositol-1,4,5-triphosphate and diacylglycerol. This pathway leads to the release of NO from a healthy endothelium and leads to vasodilation [84].

Only at high doses is it possible for ACh, through M3 receptors, to cause vasoconstriction in a healthy endothelium. Conversely, in the case of impaired endothelium, M3 receptors induce an increase in intracellular Ca^2+^ concentration in VSMCs, thereby leading to the activation of calmodulin-dependent kinase, followed by phosphorylation of myosin light chain kinase and ultimately coronary constriction [84].

## 8. Microvascular Spasm

The coronary circulation consists of several hierarchical levels, beginning with the epicardial coronary arteries with a diameter greater than 500 μm, followed by pre-arterioles (100–500 μm), arterioles (<100 μm), capillaries and finally venules and veins. Pre-arterioles and arterioles together constitute the coronary microcirculation. Coronary angiography visualizes only the epicardial coronary arteries, while the microcirculation remains beyond its resolution [86].

Coronary microvascular spasm clinically manifests as microvascular angina [87], which is characterized by typical anginal chest pain. However, symptoms may also be atypical or present as angina equivalents such as shortness of breath. Chest pain may differ in duration or character, sometimes presenting as prolonged discomfort or stabbing pain [88].

The mechanisms underlying coronary microvascular dysfunction (CMD) involve both functional and structural abnormalities of the coronary microcirculation. These mechanisms share similarities with those responsible for epicardial coronary vasospasm and include endothelial dysfunction, abnormal VSMCs contraction and inflammation [88]. Ultimately, CMD results from either impaired vasodilation and/or increased vasoconstriction within the coronary microcirculation [89].

### 8.1. Endothelial Dysfunction

Endothelial cells regulate vascular tone through the production of vasodilators such as NO, endothelium-dependent hyperpolarizing factors (EDHF) and prostaglandins, as well as vasoconstrictors including endothelin-1 (ET-1) [90].

While NO is the principal vasodilator in epicardial coronary arteries, EDHF plays a dominant role in the coronary microcirculation [22,91]. Among EDHFs, endothelium-derived hydrogen peroxide (H_2_O_2_) is considered particularly important [91,92].

EDHF, particularly H_2_O_2_, induces vasodilation by activating potassium channels and causing hyperpolarization of VSMCs membranes. The production of EDHF is stimulated by increased intracellular Ca^2+^ levels within endothelial cells [92].

Experimental studies have demonstrated that H_2_O_2_ may exert dual vascular effects, acting as both a vasodilator and a vasoconstrictor when membrane hyperpolarization mechanisms are impaired, as observed in mouse mesenteric arteries [35].

In endothelial dysfunction affecting pre-arterioles and arterioles, the balance between vasodilators and vasoconstrictors becomes disrupted. Specifically, there is reduced production of vasodilators such as H_2_O_2_ and increased production of vasoconstrictors such as ET-1 [93,94].

Endothelin-1 induces vasoconstriction by increasing intracellular Ca^2+^ concentration via activation of the phospholipase C signaling pathway and by inhibiting MLCP through activation of the ROCK pathway [36].

### 8.2. Rho-Kinase

Although the mechanisms of ROCK signaling have been discussed earlier in this review, it is important to note that ROCK activity is increased in patients with vasospastic angina who also exhibit elevated coronary microvascular resistance [17].

### 8.3. Inflammation

Chronic low-grade vascular inflammation and oxidative stress, primarily resulting from increased production of ROS, play an important role in the development of coronary microvascular dysfunction. One of the key mechanisms involves activation of the NOX system, which increases ROS production through enhanced phosphorylation of the pro-apoptotic mitochondrial protein p66Shc [37,48].

## 9. Provocation Tests

In patients with suspected CAV, particularly those presenting with vasospastic angina and non-obstructive coronary arteries, intracoronary pharmacological provocation testing with acetylcholine (ACh) and ergonovine (ER) remains the current gold standard for diagnosis. Both agents induce vasoconstriction through distinct molecular and cellular mechanisms.

As previously described, ACh acts predominantly through muscarinic (M_3_) receptors. In a healthy endothelium, M_3_ receptor activation stimulates endothelial nitric oxide synthase (eNOS) to produce nitric oxide (NO), inducing vasodilation. However, in the presence of endothelial dysfunction, this protective pathway is impaired, allowing ACh to act directly on vascular smooth muscle cells (VSMCs), triggering an increase in intracellular Ca^2+^ and subsequent vasoconstriction. Thus, ACh testing serves as a primary indicator of endothelial dysfunction. Conversely, ER exerts its effects independently of the endothelium through serotonergic (5-HT_2_) and α_1_-adrenergic receptors mediated pathways, directly activating the Rho-kinase pathway in VSMCs, which leads to hyperreactivity and smooth muscle contraction [5,95].

A positive coronary spasm provocation test is defined by transient luminal narrowing of more than 90% accompanied by ischemic electrocardiographic changes. Symptoms such as chest pain are not mandatory for the diagnosis and may be absent during testing. Coronary spasm may manifest as either focal or diffuse vasoconstriction. Focal spasm is defined as transient vessel narrowing greater than 90% localized to a major coronary artery or its branches. Diffuse spasm is diagnosed when transient vessel narrowing of ≥90% compared with baseline coronary angiography extends from the proximal to the distal segment of a major coronary artery and/or its branches [95,96].

Interestingly, ACh more commonly induces diffuse and distal coronary spasm, whereas ER more frequently provokes focal and proximal vasoconstriction, reflecting the heterogeneous distribution of endothelial injury versus localized smooth muscle hyperreactivity along the coronary vasculature [95].

The two tests are considered complementary, as some patients may exhibit a positive response to only one pharmacological agent. Acetylcholine testing is generally recommended as the initial provocation test. If the result is negative but clinical suspicion of CAV remains high, subsequent ergonovine testing should be considered to improve diagnostic yield [95].

Several studies have demonstrated high diagnostic accuracy and an acceptable safety profile when these tests are performed in experienced centers using standardized protocols, supporting their central role in the diagnostic evaluation of coronary vasomotor disorders [96,97].

As both provocation tests primarily assess epicardial coronary vasospasm, evaluation of coronary microvascular function requires invasive functional assessment using parameters such as coronary flow reserve (CFR) and the index of microcirculatory resistance (IMR). CFR and IMR are widely used invasive markers of coronary microvascular dysfunction, with reduced CFR (<2.0–2.5) and elevated IMR ≥ 25 indicating impaired microvascular function [98]. According to the Coronary Vasomotion Disorders International Study Group (COVADIS) criteria, the diagnosis of coronary microvascular dysfunction (CMD) requires the presence of anginal symptoms, the absence of obstructive coronary artery disease and objective evidence of coronary microvascular dysfunction, including reduced CFR, elevated IMR, or microvascular spasm during ACh provocation testing [88].

Furthermore, ACh testing may help differentiate epicardial coronary spasm from microvascular spasm. Microvascular spasm is characterized by reproduction of symptoms and ischemic electrocardiographic changes in the absence of significant epicardial coronary constriction during provocation testing, likely driven by downstream microvascular endothelial disruption or cellular ion-channel alterations [88].

## 10. Conclusions

The increasing prevalence of myocardial ischemia despite angiographically normal coronary arteries underscores that CAV is a complex network of interconnected pathophysiological drivers rather than a standalone phenomenon. Deciphering these cellular mechanisms—spanning oxidative stress, endothelial dysfunction, enhanced RhoA/ROCK activity and perivascular inflammatory signalling is fundamental for shifting from broad-spectrum symptom management to targeted, mechanism-based therapies.

Future diagnostic strategies must move toward mechanism-based phenotyping by integrating clinical functional testing with novel noninvasive tools. Specifically, quantifying circulating Rho-kinase activity and assessing perivascular adipose tissue (PVAT) inflammation via CT-derived pericoronary fat attenuation index (FAI) offer promising pathways for identifying distinct vasospastic endotypes.

Ultimately, prospective, mechanism-oriented clinical trials are essential to determine whether pathways-specific interventions, such as ROCK inhibition and targeted anti-inflammatory therapies, can improve long-term clinical outcomes compared with conventional strategies. Transitioning to this precision medicine paradigm represents the next frontier in coronary vasomotor dysfunction, offering the potential to reduce myocardial infarction, prevent malignant arrhythmias and alleviate the persistent anginal symptoms that severely impair quality of life in this growing patient population.

## Data Availability

No new data were created or analyzed in this study. The data discussed in this review were derived from previously published studies available in publicly accessible databases, including PubMed and Google Scholar. Therefore, data sharing is not applicable to this article.

## Abbreviations

The following abbreviations are used in this manuscript:

Ang II	Angiotensin II
Ach	Acetylcholine
CAD	Coronary Artery Disease
Ca^2+^	Calcium Ion
CAV	Coronary Artery Vasospasm
CFR	Coronary Flow Reserve
cGMP	Cyclic Guanosine 3′,5′-Monophosphate
CMD	Coronary Microvascular Dysfunction
CRP	C-Reactive Protein
CT	Computed Tomography
DNA	Deoxyribonucleic Acid
EDHF	Endothelium-Derived Hyperpolarizing Factor
eNOS	Endothelial Nitric Oxide Synthase
ET-1	Endothelin-1
ER	Ergonovine
FAI	Fat Attenuation Index
FDG	Fluorodeoxyglucose
GTP	Guanosine 5′-Triphosphate
H_2_O_2_	Hydrogen Peroxide
IMR	Index Of Microcirculatory Resistance
IL-1β	Interleukin-1β
IL-6	Interleukin-6
IκB	Inhibitor Of Nuclear Factor κB
LDL	Low-density lipoprotein
MCP-1	Monocyte Chemoattractant Protein-1
mRNA	messenger Ribonucleic Acid
MLC	Myosin Light Chain
MLCK	Myosin Light Chain Kinase
MLCP	Myosin Light Chain Phosphatase
NOX	Nicotinamide Adenine Dinucleotide Phosphate Oxidase
NO	Nitric Oxide
NF-κB	Nuclear Factor κB
PCVF	Perivascular Contractile Factor
PET	Positron Emission Tomography
PKC	Protein Kinase C
PKG	cGMP-Dependent Protein Kinase
PVAT	Perivascular Adipose Tissue
PVRF	Perivascular Relaxing Factor
p50	NF-κB p50 Subunit
p65	NF-κB p65 Subunit
ROCK	Rho-Kinase
ROS	Reactive Oxygen Species
sICAM-1	Soluble Intercellular Adhesion Molecule-1
sVCAM-1	Soluble Vascular Cell Adhesion Molecule-1
sGC	Soluble Guanylate Cyclase
VSMC	Vascular Smooth Muscle Cell
5-HT_2_	5-hydroxytryptamine receptor 2
